# Antidiarrhoeal Activity of *Musa paradisiaca* Sap in Wistar Rats

**DOI:** 10.1155/2015/683726

**Published:** 2015-03-29

**Authors:** Musa T. Yakubu, Quadri O. Nurudeen, Saoban S. Salimon, Monsurat O. Yakubu, Rukayat O. Jimoh, Mikhail O. Nafiu, Musbau A. Akanji, Adenike T. Oladiji, Felicia E. Williams

**Affiliations:** ^1^Phytomedicine, Toxicology and Reproductive Biochemistry Research Laboratory, Department of Biochemistry, University of Ilorin, PMB 1515, Ilorin 24003, Nigeria; ^2^Department of Nursing Services, University of Ilorin Teaching Hospital, PMB 1459, Ilorin 240102, Nigeria; ^3^Department of Clinical Pharmacy and Pharmacy Practice, University of Ilorin, PMB 1515, Ilorin 24003, Nigeria

## Abstract

The folkloric claim of *Musa paradisiaca* sap in the management of diarrhoea is yet to be substantiated or refuted with scientific data. Therefore, the aim of the current study was to screen the sap of *M. paradisiaca* for both its secondary metabolites and antidiarrhoeal activity at 0.25, 0.50, and 1.00 mL in rats. Secondary metabolites were screened using standard methods while the antidiarrhoeal activity was done by adopting the castor oil-induced diarrhoeal, castor oil-induced enteropooling, and gastrointestinal motility models. The sap contained flavonoids, phenolics, saponins, alkaloids, tannins, and steroids while cardiac glycosides, anthraquinones, triterpenes, cardenolides, and dienolides were not detected. In the castor oil-induced diarrhoeal model, the sap significantly (*P* < 0.05) prolonged the onset time of diarrhoea, decreased the number, fresh weight, and water content of feaces, and increased the inhibition of defecations. Na^+^-K^+^-ATPase activity in the small intestine increased significantly whereas nitric oxide content decreased. The decreases in the masses and volumes of intestinal fluid by the sap were accompanied by increase in inhibition of intestinal fluid content in the enteropooling model. The sap decreased the charcoal meal transit in the gastrointestinal motility model. In all the models, the 1.00 mL of the sap produced changes that compared well with the reference drugs. Overall, the antidiarrhoeal activity of *Musa paradisiaca* sap attributed to the presence of alkaloids, phenolics, flavonoids, and/or saponins which may involve, among others, enhancing fluid and electrolyte absorption through *de novo* synthesis of the sodium potassium ATPase and/or reduced nitric oxide levels.

## 1. Introduction

Diarrhoea is an alteration in the normal bowel movement characterized by an increase in the volume, fluidity, frequency, and passage of loose or watery stool for at least three times a day [1]. It is a common symptom of gastrointestinal infection due to ingestion of many bacteria, viruses, or parasites that may be transmitted by water, food, utensils, hands, and flies [1]. It is the mechanism by which the body rids itself of pathogenic organisms, with excessive stimulation of intestinal motility, leaving insufficient time for absorption of intestinal fluid [2]. Diarrhoea is one of the most important health problems in the developing countries affecting people of all ages [3] that results in electrolyte loss, dehydration, shock, and sometimes death [1]. Diarrhoeal diseases account for 1 in 9 child deaths worldwide, making diarrhoea the second leading cause of death among children under the age of 5 [4]. In Nigeria, the prevalence of diarrhoeal infection is as high as 18.8%, above the average of 16%, making it one of the worst in Sub-Saharan Africa [5]. It accounts for an annual estimated 300,000 deaths mainly amongst children under five in Nigeria [5], while 7-to-12-month-old babies continue to be the most susceptible [6] caused mainly by poor sanitation and hygiene practices. The disease may be caused by a wide array of agents such as enteropathogenic microorganisms (*Shigella flexneri*,* Staphylococcus aureus*,* Escherichia coli*,* Salmonella typhi,* and* Candida albicans*) [7], alcohol, irritable bowel syndrome, bile salts and hormones [8], secretory tumors, and intoxication [9]. Several options employed in the management of the diseases include oral agents such as metronidazole, antibiotics, and oral rehydration therapy (ORT) [10, 11]. With over a decade of the practice and promotion of ORT, diarrhoea is still the second among the causes of child death [12]. In addition, these management options which often fail during high stool output state are also associated with undesirable side effects such as headache, convulsion, stomach cramp, vomiting, constipation, and hallucination. Consequently, attention is now being shifted to alternatives in medicinal plants for the management of the disease. From the ethnobotanical survey carried out by the authors and literature search, the sap of* M. paradisiaca* was mentioned as a plant part frequently used in managing diarrhoea.

*Musa paradisiaca* (Musaceae), also commonly called plantain (English),* “ogede agbagba”* (Yoruba),* “ayabar turawa”* (Hausa), and* “ogadejioke”* (Igbo), is native to South East Asia and India but is now extensively cultivated in the tropical and subtropical regions.* M. paradisiaca* is a herbaceous plant of up to 9 m long. The pseudostem (consisting of leaves and their fused bases) of plantain grows up to 7–20 inches with a crown of large elongated oval deep-green leaves (up to 365 cm in length and 61 cm in width), with a prominent midrib. It has long barbs of green-whitish flowers. The female flowers appear further up the stem and produce the actual fruit without fertilization. The leaves are ovate and long and can be distinguished by their chunky footstalk.* M. paradisiaca* produces green or greenish-yellow seedless fruits that are oblong, fleshy, and 7 cm long in wild form and longer in the cultivated varieties. Each plant produces a single inflorescence like drooping spike and large bracts opening in succession [13].

Various parts of the plant have been claimed to be ethnopharmacologically relevant in the management of several ailments. For example, a cold infusion of the root is used to treat venereal diseases, anaemia, scabies, leprosy, and skin diseases [14]. The fruit is consumed as food and used as tonic, antihelminthic, depurative, diuretic, emollient, antiscorbutic, and aphrodisiac, while the leafy juice was reported to be used in the treatment of fresh wounds, cuts, insect and snake bites [15]. The leaves have also been used for managing cold, bronchitis, and eye infections and as an abortifacient. Furthermore,* M. paradisiaca* sap has also been claimed to be used as a remedy for diarrhoea, dysentery, hysteria, and epilepsy [15]. The ethanolic and ethanol : water (1 : 1) extracts of* M. paradisiaca* flower have been reported to exhibit antimicrobial activity with minimum inhibitory concentrations ranging from 5.62 to 25.81 and 7.60 to 31.50 *μ*g/mL, respectively [16]. The extract of plantain at 50 mg/kg, administered twice daily for 5 days, has also been reported to have significant antiulcer effect and antioxidant activity in gastric mucosal homogenates of rats [17]. On the antihyperglycemic activity, the methanolic extract of* M. paradisiaca* green fruits (100–800 mg/kg p.o.) induced significant, dose-related reductions in the blood glucose concentrations of both normal and diabetic mice [18]. Agarwal et al. [19] attributed the wound healing activity of the aqueous and methanolic extract of unripe* M. paradisiaca* fruit (50, 100, and 200 mg/kg) to its antioxidant property. The aqueous extract of* M. paradisiaca* leaves at 250 and 1000 mg/kg has also been reported to possess analgesic activity in hot plate reaction time and acetic acid-induced writhing models [20] whereas the alcoholic extract at the doses of 250 and 500 mg/kg, p.o., and aqueous extract of the stem of* M. paradisiaca* at a dose of 500 mg/kg, p.o., have been reported to have significant effect on the liver of CCl_4_ and paracetamol-induced hepatotoxicity animal models [21]. The galactogogue effects of* M. paradisiaca* flower extract in lactating rats [22], haemostatic effect of* M. paradisiaca* stem juice in Guinea pigs [23], and the physicochemical properties and fatty acid composition of the oil from ripe plantain peel (*Musa paradisiaca*) [24] have all been reported.

Despite several scientific studies that have validated the folkloric uses of different parts of* M. paradisiaca,* there is no study in the open scientific literature that has provided scientific evidence to the acclaimed use of the sap of* M. paradisiaca* in the management of diarrhoea. Therefore, this study was aimed at validating or not, the acclaimed use of* M. paradisiaca* sap in the management of diarrhoea using chemically induced diarrhoeal models in rats.

## 2. Materials and Methods

### 2.1. Plant Material

The plant, obtained from a plantation (Baba Noble Plantation), Tanke, Ilorin, Kwara State, Nigeria, was authenticated by Mr. Onadeji Michael of the Forestry Research Institute of Nigeria (FRIN), Ibadan, Nigeria. A voucher specimen assigned with FHI 10846 was deposited in the herbarium of the Institute of FRIN.

### 2.2. Animals

Ninety, healthy Wistar rats (126.77 ± 8.11 g) were obtained from the animal house of the Department of Biochemistry, University of Ilorin, Ilorin, Nigeria. All the animals were housed in clean plastic cages that were placed in a well-ventilated house (temperature: 22 ± 3°C; photoperiod: 12 hours; humidity: 45–50%). The animals were fed on rat pellets (Premier Feed Mill Co. Ltd., Ibadan, Nigeria) and clean tap water, except when fasting was required during the experiment. The cages and the animal house were cleaned on daily basis.

### 2.3. Drugs and Chemicals

Atropine sulphate and loperamide hydrochloride were products of Laborate Pharmaceuticals (India) and Richy Gold International Limited (Nigeria), respectively. Castor oil was a product of Bills Sons and Co. (Druggist) Limited, Southport, England.

### 2.4. Collection of* Musa paradisiaca* Sap

The sap of* Musa paradisiaca* was obtained by slantingly cutting the internode of the plant and allowing the sap to flow freely into a clean sterilized conical flask. The sap was used within six hours of collection.

### 2.5. Qualitative Screening of Secondary Plant Metabolites

A known volume 1.0 mL of the sap of* M. paradisiaca* was screened for the presence of some secondary metabolites as described for alkaloids [25], steroids, anthraquinones, cardenolides and dienolides [26], saponins [27], phenolics and flavonoids [28], cardiac glycosides [29], tannins and triterpenes [30] as follows.

*Saponins*. (frothing test) A known volume (2.0 mL) of the sap was boiled in 20 mL of distilled water in a water bath and filtered. The filtrate (10.0 mL) was mixed with 5.0 mL of distilled water and shaken vigorously for a stable persistent froth which confirms the presence of saponins.

*Alkaloids*. Exactly 1.0 mL of the sap was stirred with 5.0 mL of 1% v/v aqueous HCl on a steam bath and filtered while hot. Distilled water was added to the residue and 1.0 mL of the filtrate was treated with two drops of Mayer's reagent (potassium mercuric iodide solution), Wagner's reagent (solution of iodine in potassium iodide), and Dragendorff's reagent (solution of potassium bismuth iodide). The formation of a cream colour with Mayer's reagent, reddish-brown precipitate with Wagner's and Dragendorff's reagents give a positive test for alkaloids.

*Phenolics*. Two drops of 5% w/v of FeCl_3_ was added to 1.0 mL of the plant sap. The appearance of a greenish precipitate indicated the presence of phenolics.

*Cardiac Glycosides*. 1.0 mL of the sap was added to 2.0 mL of chloroform. Thereafter, 2.0 mL of H_2_SO_4_ was carefully added. A reddish-brown colour at the interface indicated the presence of aglycone portion of cardiac glycosides.

*Tannins*. 1.0 mL of freshly prepared 10% w/v ethanolic KOH was added to 1.0 mL of the sap. A white precipitate indicated the presence of tannins.

*Steroids*. Five drops of concentrated H_2_SO_4_ was added to 1.0 mL of the sap. The appearance of red colour indicated the presence of steroids.

*Triterpenes.* A known volume (1.0 mL) of the sap was added to 5 drops of acetic acid anhydride followed by a drop of concentrated H_2_SO_4_. The mixture was steamed for 1 hour and neutralized with NaOH followed by the addition of chloroform. Appearance of bluish-green colour indicated the presence of triterpenes.

*Anthraquinones*. Exactly 3.0 mL of the sap was shaken with 10.0 mL of benzene and filtered, afterwhich 5.0 mL of 10% v/v NH_4_OH was added to the filtrate. The appearance of a pink colour in the ammonical (lower) phase indicated the presence of anthraquinones.

*Flavonoids*. Exactly 3.0 mL of the filtrate was mixed with 4.0 mL of 1% potassium hydroxide in a test tube. A dark yellow colour indicated the presence of flavonoids.

*Cardenolides and Dienolides*. A portion (5.0 mL) of the sap was added to 2.0 mL of glacial acetic acid containing one drop of 5% w/v FeCl_3_ solution. This was then followed by the addition of 1.0 mL of concentrated H_2_SO_4_. A brown ring at the interface indicated the presence of a deoxy sugar characteristic of cardenolides.

### 2.6. Castor Oil-Induced Diarrhoea in Rats

Thirty, healthy Wistar rats were fasted for 6 hours prior to the experiment but allowed free access to water. The experimental rats were completely randomized into five groups of six animals each. The procedure described by Bajad et al. [31] was adopted with slight modification. Animals in group A (administered 1.0 mL of distilled water) served as negative control, while those in groups B (positive control), C, D, and E (test groups) received 1.0 mL each corresponding to 2.5 mg/kg body weight of loperamide (a reference drug), 0.25, 0.5, and 1.0 mL of the sap, respectively. Thirty minutes after administration, all the animals were orally administered 1 mL of castor oil and thereafter placed in cages lined with a preweighed transparent paper. During the 6-hour observation period, the time of onset of diarrhoea, the total number of faeces, diarrhoeal faeces, total weight of faeces, and percentage inhibition of diarrhoeal defecation in each group were computed. The weight of the faeces was obtained from the difference in the preweighed transparent paper and fresh weight of the stool. The dry weight of the faeces was obtained by drying the fresh faeces in the laboratory oven (Uniscope Laboratory oven, SM9053, Surgifriend Medicals, England) at 100°C until constant weight was obtained. The difference in the fresh weight of the faeces and dry weight of the faeces gives the water content of the faeces. At the end of the 6-hour exposure period, the animals were sacrificed and small intestine supernatants prepared.

### 2.7. Preparation of Small Intestine Supernatants

The procedure described by Akanji and Yakubu [32] was adopted for the preparation of small intestine supernatants. Briefly, under ether anaesthesia, the animals were dissected and small intestine was removed. Thereafter, the contents of the small intestines were squeezed out, blotted in blotting paper, and homogenized in 0.25 M sucrose solution (1 : 4 w/v) using Teflon homogenizer. The homogenate was centrifuged at 894 ×*g* for 15 minutes to obtain the supernatant which was used for the determination of concentrations of protein, nitric oxide, and the activity of sodium potassium ATPase.

### 2.8. Determination of Protein Content of the Supernatant

The protein concentration in the small intestine supernatant of the animals was assayed, using Biuret reagent as described by Gornall et al. [33]. A known volume (4.0 mL) of Biuret reagent was added to 1.0 mL of the supernatant (appropriately diluted). This was mixed thoroughly by shaking and left undisturbed for 30 minutes at room temperature for colour development. The blank was constituted by replacing the supernatants with 1.0 mL of distilled water. The absorbance was read against blank at 540 nm. The concentration of protein in the supernatants was obtained from the calibration curve for bovine serum albumin using the expression

(1) $$\begin{array}{c} \text{Protein}\;\text{concentration}\left(\text{mg}/\text{mL}\right)=C_{s}\times F, \end{array}$$

where *C*_*s*_ = corresponding protein concentration from the calibration curve; *F* = dilution factor.

### 2.9. Determination of Na^+^-K^+^ ATPase Activity

The procedure described by Bewaji et al. [34] was adopted for the determination of the activity of Na^+^-K^+^ ATPase in the small intestine supernatant. Briefly, 400 *μ*L of 200 mM of NaCl, 40 mM of KCl, and 60 mM of Tris (pH 7.4) were pipetted into test tube. Thereafter, 20 *μ*L of MgCl_2_·6H_2_O (80 mM), 20 *μ*L of 20 mM EGTA, 240 *μ*L of distilled water, and 20 *μ*L of appropriately diluted supernatant of the small intestine were added. The mixture was incubated at 37°C for 5 minutes. A known volume (100 *μ*L) of 8 mM ATP was added, mixed thoroughly, and incubated at 37°C for 30 minutes. Furthermore, 200 *μ*L of sodium dodecyl sulphate (5%) and 2,000 *μ*L of reagent C (mixture of ammonium molybdate/sulphuric acid solution [reagent A] and 9% ascorbic acid [reagent B] in ratio 4 : 1 v/v) were added. The mixture was left undisturbed for 30 minutes at room temperature for colour development. The blank was constituted in the same manner except that the small intestine supernatant was replaced with 20 *μ*L of distilled water. The absorbance of the test solution was read against that of the blank at 820 nm and then extrapolated from the absorbance obtained was then extrapolated from the calibration curve for phosphate to give the concentration of the inorganic phosphate. Thereafter, the specific activity of Na^+^-K^+^ ATPase was computed using the following expression:

(2) Specific  activity(μmole  Pi/mg  protein/hr)   =[Pi]×2×dilution  factor1000×protein  concentration(mg/mL),

where [P_*i*_] = concentration of inorganic phosphate in nmoles (obtained from the calibration curve); 2 = factor introduced to obtain the amount of P_*i*_ released per hour; 1000 = factor introduced to convert the P_*i*_ released to *μ*moles.

### 2.10. Determination of Nitric Oxide Concentration

The procedure described by Wo et al. [35] was used to determine the concentration of nitric oxide in the small intestine supernatants of the animals. A known volume (0.5 mL) of the supernatant was added to 2 mL of 75 mmol/L ZnSO_4_ solution and 2.5 mL of 55 mmol/L NaOH. The solution was mixed thoroughly, adjusted to a pH of 7.3, incubated for 10 minutes, and centrifuged at 504 ×g for 10 minutes. The blank was constituted in a similar manner like the test except that 0.5 mL of supernatant was replaced by 0.5 mL of distilled water. Furthermore, 1 mL of glycine-NaOH buffer each was added to the test sample and blank. A known volume (2 mL) of deproteinized solution was added to the test and blank and the volume adjusted to 4.0 mL with deionized distilled water. The reaction was initiated by the addition of freshly activated cadmium granules and, after 60 minutes, 2.0 mL each of test and blank was added to tubes containing 2.5 mL of ethylenediaminetetraacetic acid solution, 3.0 mL of 1.0 mol/L HCl, and 0.3 mL of 1.0 g/L fuchsin acid solution, mixed thoroughly and incubated for 2 minutes. Next, 0.2 mL of 0.05 mol/L resorcinol and 3.0 mL of 1.0 mol/L NH_4_OH were added. The absorbance of the test solution was read against the blank at 436 nm. The concentration of serum nitrite was extrapolated from the calibration curve of nitrite.

### 2.11. Castor Oil-Induced Enteropooling

The procedure described by Chitme et al. [36] was adopted for the castor oil-induced enteropooling study. The animals were fasted without food for 6 h prior to the experiment but were allowed free access to water. Six animals were randomly selected for each group and were placed in their respective cages. Animals in the negative control group (group A) received 1.0 mL of distilled water, while those in the positive control group (group B) received 1.0 mL of atropine sulphate (2.5 mg/kg body weight). Rats in groups C, D, and E (test groups) were orally administered 0.25, 0.50, and 1.0 mL of the sap, respectively. Immediately afterwards, 1.0 mL of castor oil was administered orally to each of the rats in all the groups. After 30 minutes, each rat was sacrificed according to the method described by Akanji and Yakubu [32] and the ends of the pylorus and caecum of the small intestine were tied. The small intestine was dissected and its intestinal content squeezed into a measuring cylinder. The volumes and the masses of the intestinal contents were noted and used to compute the percentage of inhibition of intestinal content.

### 2.12. Gastrointestinal Motility

The method described by Teke et al. [8] was adopted for the evaluation of the effect of the sap on gastrointestinal transit in the rats. Thirty, healthy Wistar rats were fasted for 6 hours prior to the experiment but were allowed free access to water. The experimental rats were completely randomized into five groups of six animals each. The negative control group (group A) received 1.0 mL of distilled water while the positive control group (group A) received 1.0 mL of atropine sulphate (0.6 mg/mL) intramuscularly. Animals in groups C, D, and E (test groups) were orally administered 0.25, 0.50, and 1.0 mL of the sap, respectively. Charcoal meal (10% charcoal suspension in 5% agarose agar, prepared by weighing 10 g of charcoal powder and 5 g of agarose agar into 100 mL distilled water and mixed thoroughly) was administered orally, 30 minutes after the administration of atropine sulphate and the sap of* M. paradisiaca*. The animals were then sacrificed after 45 minutes of charcoal administration, using the diethyl ether as anesthesia as described by Akanji and Yakubu [32]. The small intestine was removed very carefully and the length of the intestine as well as distance traveled by the charcoal meal through the intestine was measured. The percentage of gastrointestinal motility was computed as the ratio of distance moved by the charcoal meal to the length of the small intestine.

### 2.13. Ethical Consideration

The animals were humanely handled according to the guidelines of the European Convention for the protection of vertebrate animals and other scientific purposes, ETS-123 [37].

### 2.14. Statistical Analysis

Data were expressed as the mean ± SEM of six determinations. Mean was analyzed using analysis of variance and complemented with Tukey's post hoc test. Statistical Package for Social Sciences, version 18.0 (SPSS Inc., Chicago, IL, USA), was used to carry out the statistical analyses. Statistical significance was set at 95% confidence interval [38].

## 3. Results

**Figure 1:**
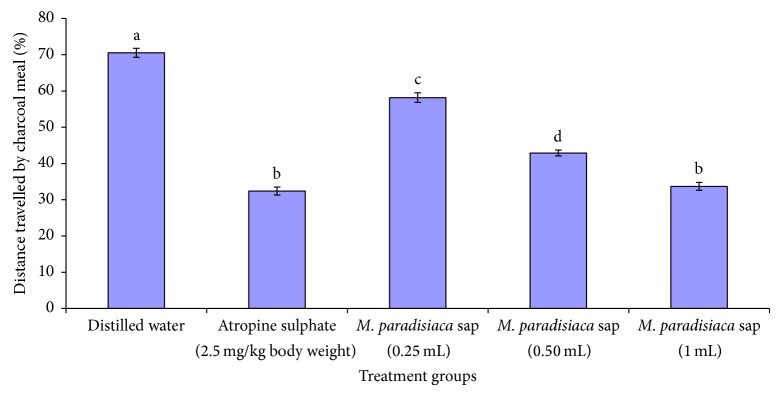
Effect of* Musa paradisiaca* sap on charcoal meal transit time of Wistar rats.

The analysis of secondary metabolite constituents of* M. paradisiaca* sap revealed the presence of alkaloids, saponins, flavonoids, tannins, phenolics, and steroids, while anthraquinones, triterpenes, cardiac glycosides, cardenolides, and dienolides were not detected (Table 1). In the castor oil-induced diarrhoeal experiment, the sap of* M. paradisiaca* significantly (*P* < 0.05) and dose-dependently prolonged the time of onset of diarrhoea. In addition, the number of wet feaces, total number of feaces, and fresh weight and water content of the faeces decreased significantly (*P* < 0.05) (Table 2). The reductions exhibited by the 1.0 mL of the sap were more than the other dose levels and also compared favourably (*P* > 0.05) with those administered the reference drug, loperamide. Furthermore, the computed inhibition of defecation increased in a dose-dependent manner with the most remarkable inhibition occurring at 1.0 mL of the sap; this also compared well with the castor oil-induced diarrhoeal rats treated with loperamide (Table 2). The activity of sodium potassium ATPase in the small intestine increased significantly (*P* < 0.05) in a dose-dependent manner following the administration of the sap, whereas the sap significantly (*P* < 0.05) and dose-dependently decreased the concentration of nitric oxide (Table 2). It is worthy of note that the reduction in nitric oxide by the 0.25 mL of* M. paradisiaca* sap compared well with that of loperamide treated animals (Table 2). The sap significantly (*P* < 0.05) and dose-dependently decreased the masses and volumes of the intestinal fluid in the castor oil-induced enteropooling in Wistar rats (Table 3). In contrast, the inhibitions of the intestinal contents of the animals were increased by the sap in a dose-related manner (Table 3) with that of 1.0 mL producing the most remarkable inhibition of all the treatment groups (Table 3). In the charcoal meal transit time, the sap dose-dependently reduced the transit time of the charcoal meal during the 45-minute exposure period (Figure 1).

## 4. Discussion

*M. paradisiaca* sap has been traditionally acclaimed to be used in the management of diarrhoea without any scientific study that has validated or refuted this claim. Therefore, in the present study, the traditionally acclaimed use of* M. paradisiaca* sap in the management of diarrhoea was substantiated with scientific evidence using chemically-induced diarrhoea models such as castor oil-induced diarrhoea, castor oil-induced enteropooling, and charcoal meal gastrointestinal motility.

Agents that cause diarrhoea do this through several mechanisms such as active electrolyte secretion, decreased electrolyte absorption, increased luminal osmolality, changes in mucosal morphology and permeability, and disordered motor activity. Castor oil induces diarrhoea in experimental animals and humans through its active metabolite, ricinoleic acid. Ricinoleic acid causes diarrhoea through a series of events which include stimulating the peristaltic activity of the small intestine and reducing and/or inhibiting the activity of Na^+^-K^+^ ATPase. These consequently lead to changes in the electrolyte permeability of the intestinal mucosa, hypersecretion of the intestinal contents, and a slowdown of the transport time in the intestine [39, 40]. Furthermore, the action of ricinoleic acid as diarrhoeic agent might suggest an influence on the nitric oxide/prostaglandin pathway with the consequent release of endogenous mediators such as nitric oxide and prostaglandin. Endogenous nitric oxide during physiological condition elicits diarrhoea by acting as proabsorptive molecule whereas, in the pathophysiological states, the elevated levels of NO may provoke net secretion of electrolytes [41]. On the other hand, prostaglandin causes diarrhoea by stimulating both the intestinal electrolyte and fluid secretion and inhibiting sodium absorption [42]. Therefore, the prolonged time of induction of diarrhoea, decreased frequency of stool (total number of feaces), number of wet feaces, fresh weight of feaces, and water content of feaces by the sap of* M. paradisiaca* not only is suggestive of antidiarrhoeal action but might explain the rationale for its sustained use in folk medicine as an antidiarrhoeal agent in the animals. The antidiarrhoeal activity of* M. paradisiaca* sap was supported by the increase in the computed inhibition of defecation. Na^+^-K^+^ ATPase plays an important role in the absorption of sodium and fluid in the intestine of the animals. Thus, inhibition of this intestinal enzyme may contribute to intestinal fluid accumulation which consequently can predispose the animals to diarrhoea [43]. The dose-dependent increase in Na^+^-K^+^ ATPase by the sap of* M. paradisiaca* suggests that the accumulation of fluids in the intestine might have been impaired and this consistently emphasizes the antidiarrhoeal activity of the sap from* M. paradisiaca*. In contrast, the reduction in the levels of intestinal nitric oxide by the sap might be an indication that the net secretion of the electrolytes was not enhanced. It is worthy of note that all the changes in the fecal parameters, activity of Na^+^-K^+^ ATPase, and the level of nitric oxide by the sap of* M. paradisiaca* were similar and statistically comparable to those of the reference drug, loperamide. Loperamide apart from regulating the gastrointestinal tract has also been reported to slow down small intestinal transit, reduce colonic rate of flow, and consequently increase colonic water absorption, but it does not have any effect on colonic motility [44]. Therefore, the reduction in the episode of diarrhoea by loperamide may be attributed to any of these aforementioned mechanisms. It is also important to note that the most profound evidence in support of the antidiarrhoeal activity was exhibited by the 1.0 mL of the sap. Since castor oil produces diarrhoea by preventing fluid and electrolyte absorption and thus increasing the intestinal peristaltic movements [45], it may therefore be proposed that the mechanism of action of* M. paradisiaca* sap as antidiarrhoeal agent may be to enhance fluid and electrolyte absorption through the gastrointestinal tract and enhance the activity of Na^+^-K^+^ ATPase through its* de novo* synthesis or might have an influence on the NO/prostaglandin pathway. The exact mechanism may have to await the outcome of further research.

Atropine sulphate produces diarrhoea through anticholinergic effect on intestinal transit thereby increasing the gastrointestinal motility [46] whereas activated charcoal prevents the absorption of drugs and other chemicals into the body [47]. Therefore, the inhibition of castor oil-induced intestinal fluid accumulation (enteropooling) and the weight of the intestinal content as well as the suppressed intestinal propulsive movement of the charcoal meal by the sap of* M. paradisiaca* during the 45 minutes exposure period suggest significant inhibition of gastrointestinal motility. This further indicates that the sap is capable of preventing cholinergic transmission or its anticholinergic effect on the gastric mucosa [48]. It also shows that the sap increased the time needed for the absorption of water and electrolytes in a manner similar to that of atropine sulphate. However, since electrolyte absorption determines the efficiency of nutrient absorption [49], it is likely that the enhanced electrolyte absorption by the sap may have stimulated the absorption of other intestinal contents. The findings in this study with respect to enteropooling and charcoal meal transit time are similar to those reported by Shaphiullah et al. [48] following the administration of methanolic extract of* Ludwigia hyssopifolia* at the dose of 100 mg/kg body weight to mice.

Besides these proposed mechanisms of action of the sap as an antidiarrhoeal agent, the extract might contain some remarkable chemical compounds with high affinity for the opioid receptor on the gastrointestinal mucosa and relieves diarrhoea when activated by an agonist [45]. Some of these chemical compounds may include the secondary metabolites like flavonoids, tannins, alkaloids, saponins, reducing sugars, and sterols and/or terpenes [50–53]. Flavonoids in the sap might be responsible for the antidiarrhoeal activity by inhibiting intestinal motility and hydroelectrolytic secretion [54], while the astringency exhibited by tannins through denaturing protein constituents of the intestinal mucosa and forming protein tannates may also account for its antidiarrhoeal activity. Furthermore, saponins also exhibit antidiarrhoeal activity by inhibiting the release of histamine [55]. Therefore, the presence of any of these compounds in the sap of* M. paradisiaca* acting singly or synergistically may confer antidiarrhoeal activity on the sap.

Overall, the results of this study have shown that* Musa paradisiaca* sap possesses antidiarrhoeal activity through some or all of the proposed mechanisms with the most remarkable antidiarrhoeal activity at the 1.0 mL of the sap. The antidiarrhoeal activity is suspected to be due to the presence of secondary metabolites contained in the sap. The use of the sap of* Musa paradisiaca* in the folk medicine as a nonspecific antidiarrhoeal agent has been substantiated with scientific evidence in the present research. Further research is needed to unravel the bioactive agent(s) and its (or their) actual mechanism of action as an antidiarrhoeal agent.

## Figures and Tables

**Table 1:** Secondary metabolite constituents of *Musa paradisiaca* sap.

Secondary metabolites	Observation	Inference
Alkaloids	Cream colour with Mayer's reagent; Reddish-brown precipitate with Wagner's and Dragendorff's reagents	Present
Tannins	White precipitate with ethanolic KOH	Present
Saponins	Stable, persistent froth with water	Present
Flavonoids	Dark yellow colour with KOH	Present
Phenolics	Greenish precipitate with FeCl_3_	Present
Steroids	Red colour with drops of concentrated H_2_SO_4_	Present
Triterpenes	Absence of bluish-green colour	Not detected
Cardiac glycosides	Absence of reddish-brown colour at the interface	Not detected
Anthraquinones	Absence of pink in the ammoniacal phase	Not detected
Cardenolides and dienolides	Absence of brown ring at the interfere	Not detected

**Table 2:** Effects of *Musa paradisiaca* sap on castor oil-induced diarrhoea in Wistar rats.

	Distilled water	Loperamide (mg/kg body weight)	*Musa paradisiaca* sap (mL)		
Parameters/dose (mL)	0	2.5	0.25	0.5	1.0

Onset time (min)	63.10 ± 1.60^a^	122.30 ± 3.74^b^	72.30 ± 2.62^c^	97.40 ± 2.61^d^	134.50 ± 2.62^e^
Total number of feaces	5.11 ± 0.74^a^	2.11 ± 0.59^b^	3.89 ± 0.90^c^	2.82 ± 0.26^d^	2.30 ± 0.71^b^
Number of wet feaces	4.33 ± 0.47^a^	0.80 ± 0.12^b^	1.94 ± 0.37^c^	0.94 ± 0.13^d^	0.67 ± 0.06^e^
Fresh weight of feaces (g)	3.71 ± 0.21^a^	0.71 ± 0.05^b^	2.14 ± 0.37^c^	0.87 ± 0.13^d^	0.58 ± 0.02^b^
Water content of feaces	1.78 ± 0.25^a^	0.33 ± 0.06^b^	1.08 ± 0.23^c^	0.86 ± 0.12^c^	0.36 ± 0.04^b^
Inhibition of defecation (%)	0	58.70	23.80	44.80	54.90
Small intestine Na + K + ATPase activity (*μ*mol Pi/mg protein/hour)	831.16 ± 10.44^a^	1012.05 ± 13.68^b^	1321.04 ± 13.19^c^	1491.48 ± 9.60^d^	1610.02 ± 15.11^e^
Small intestine nitric oxide concentration (*μ*mol/L)	162.14 ± 9.02^a^	91.22 ± 7.54^b^	92.29 ± 5.36^b^	70.09 ± 7.09^c^	56.27 ± 4.30^d^

Data are mean of six determinations ± SEM. Test values with superscripts b, c, d, and e different from that of the castor oil-induced diarrhoea distilled water treated control, a, across the row for each parameter are significantly different (*P* < 0.05).

**Table 3:** Effects of *Musa paradisiaca* sap on castor oil-induced enteropooling in Wistar rats.

	Distilled water	Atropine sulphate (mg/kg body weight)	*Musa paradisiaca* sap (mL)		
Parameters/dose (mL)	0	2.5	0.25	0.5	1.0
Mass of intestinal fluid (g)	2.17 ± 0.08^a^	1.30 ± 0.09^b^	2.01 ± 0.07^a^	1.71 ± 0.07^c^	1.04 ± 0.04^d^
Volume of intestinal fluid (mL)	2.14 ± 0.05^a^	1.26 ± 0.04^b^	1.98 ± 0.06^a^	1.67 ± 0.06^c^	1.02 ± 0.02^b^
Inhibition of intestinal content (%)	0	41.12	7.47	21.90	52.30

Data are mean of six determinations ± SEM. Test values with superscripts b, c, and d different from that of the castor oil-induced diarrhoea distilled water treated control, a, across the row for each parameter are significantly different (*P* < 0.05).
